# Multi-modal Educational Curriculum to Improve Richmond Agitation-sedation Scale Inter-rater Reliability in Pediatric Patients

**DOI:** 10.1097/pq9.0000000000000096

**Published:** 2018-08-07

**Authors:** Margaret J. Kihlstrom, Ashley P. Edge, Kelly M. Cherry, Paul J. Zarick, Shawna D. Beck, Jenny M. Boyd

**Affiliations:** From the * Division of Pediatric Critical Care Medicine, Department of Pediatrics, University of North Carolina at Chapel Hill, Chapel Hill, N.C.; † Practice Quality and Innovation, University of North Carolina Health Care, Chapel Hill, N.C.; ‡ Department of Pharmacy, University of North Carolina at Chapel Hill, Chapel Hill, N.C.

## Abstract

**Introduction:**

The Richmond Agitation-sedation Scale (RASS) is a reliable and valid scale for assessing sedation in critically ill pediatric patients. This investigation evaluates the inter-rater reliability of the RASS in mechanically ventilated pediatric patients before and after an educational intervention.

**Methods:**

This prospective, interventional quality improvement study was completed in a 20-bed pediatric intensive care unit from July 2013 to July 2014. Children 0–18 years of age requiring mechanical ventilation and receiving sedative or analgesic medications were eligible. Staff completed simultaneous paired RASS assessments in 3 phases: baseline, after educational intervention, and maintenance.

**Results:**

Staff completed 347 paired assessments on 45 pediatric intensive care unit patients: 49 in the baseline phase, 228 in the postintervention phase, and 70 in the maintenance phase. There was a significant increase in the weighted κ after the intervention, from 0.56 (95% CI, 0.39–0.72) to 0.86 (95% CI, 0.77–0.95; *P* < 0.001). The improvement was maintained months later with weighted κ 0.78 (95% CI, 0.61–0.94). In subgroup analysis, there was an increase in weighted κ in patients less than 1 year of age (0.41–0.87) and those with developmental delay (0.49–0.84).

**Conclusions:**

The RASS is a reliable tool for sedation assessment in mechanically ventilated, sedated pediatric patients after implementation of an educational intervention. It is also reliable in patients less than 12 months of age and patients with developmental delay. The ability to easily educate providers to utilize a valid, reliable sedation tool is an important step toward using it to provide consistent care to optimize sedation.

## INTRODUCTION

Providing appropriate sedation and analgesia for mechanically ventilated critically ill pediatric patients is a vital aspect of their care. When delivered optimally, sedation and analgesia medications can alleviate pain and anxiety, enhance patient safety, decrease hospital length of stay, and facilitate performance of invasive procedures.^1–7^ However, no standard practice exists to assess or achieve the desired sedation level, leading to wide variation.^8–10^ Similarly, no consensus exists regarding the optimal level of sedation, with suboptimal sedation levels in many pediatric intensive care unit (PICU) patients.^1^

A multidisciplinary panel from the United Kingdom recommended the regular assessment and documentation of sedation level using a formal sedation assessment scale.^8^ The Richmond Agitation-sedation Scale (RASS) is a valid and reliable tool used for sedation assessments in adult patients.^11–18^ The RASS is becoming a common tool to evaluate sedation level in pediatric patients as supported by its inclusion as a component in 2 pediatric delirium assessment tools and a recent study by Kerson et al.,^19^ which demonstrated that it is valid and reliable in assessing sedation in critically ill pediatric patients.^20,21^ However, to date, no studies exist that explore the use of an educational intervention to improve and maintain the reliability of the scale over time in pediatric patients.

This investigation evaluates the inter-rater reliability (IRR) of the RASS in mechanically ventilated pediatric patients before and after an educational intervention in a PICU in which staff used the RASS as a sedation assessment for over 5 years. The research team chose the RASS, given its quick and easy use at the bedside compared with other scales; the staff’s prior familiarity with the scale; and its existing integration into the medical record for documentation. We hypothesized that the reliability would improve significantly with an educational intervention. Because the pediatric population is unique in that assessments of sedation and pain can be affected by young age and developmental delay,^22–25^ the reliability in these 2 groups was also specifically evaluated.

## METHODS

This prospective interventional study was conducted in the PICU at North Carolina Children’s Hospital in Chapel Hill, NC, which is a 20-bed unit with 1,200 admissions annually that include medical, surgical, and cardiac patients. The institutional review board approved the study and waived consent for patients and staff involved. IRR data were collected in 4 phases from July 7, 2013, to July 16, 2014, on patients 18 years of age or younger requiring mechanical ventilation and receiving infusions of sedative or analgesic medications. At the time of the study, no sedation protocol was in place in the PICU; the ordering provider determined the infusions. Commonly used medications included fentanyl, morphine, midazolam, and dexmedetomidine; the study rarely included ketamine and propofol. Excluded patients included those with preexisting impaired hearing, impaired vision, and those receiving neuromuscular blockade. A core research team of 2 physicians and 2 nurses performed RASS assessments with PICU nurses and physicians. Assessments were performed using a slightly adapted version of the original RASS, which Ely et al.^13^ found to be valid and reliable in adult ICU patients (Table 1). The research team decided that the criteria of eye-opening in addition to eye contact for scores −1 to −3 enhances its use in neonates who do not make eye contact developmentally. Subgroup analysis was also performed on patients less than 1 year of age and those with developmental delay, as defined by the problem list during admission.

**Table 1.**
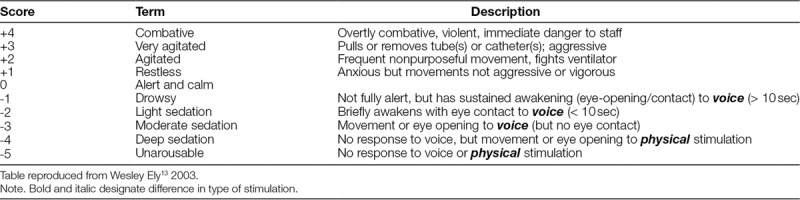
Richmond Agitation-sedation Scale

### Baseline Phase

From July 7, 2013, to September 12, 2013, assessments of RASS IRR were performed in the PICU to ascertain a baseline among unit staff. The RASS was already utilized for sedation documentation by nurses in the electronic medical record; however, they had previously received no formal education on how to utilize the tool. A member of the research team combined with a PICU nurse or physician obtained IRR measurements by performing simultaneous independent RASS assessments of mechanically ventilated patients. The team first observed the patient for any signs of agitation. If none were present, the patient was then verbally stimulated, followed by physical stimulation if there was no response to voice. A RASS score was then assigned based on the scale in Table 1. Each participant was blinded to the scores of other participants. The majority of assessments were completed between 7 am and 7 pm on weekdays due to the availability of research team members.

### Intervention Phase

From October 1, 2013, to February 23, 2014, the research team implemented multi-modal education for nurses and physicians in the PICU. Education included a 4-pronged approach: independent online modules, one-on-one in-servicing with staff, group lectures, and visual aids/reminders. Staff completed an online learning module that described the RASS and included a posttest for completion. Each bedside nurse received individual education by a member of the research team with a written script that included how and when to document the RASS and reiterated the points demonstrated in the online module. Physicians attended educational conferences conducted by research team members. Visual aids included posters of the RASS in patient rooms proximal to the computer used for documentation, a copy of the RASS to attach to hospital badges, reminders throughout the unit and common staff areas to perform and document RASS assessments, and the addition of a “Goal RASS Score” to the daily goal sheet used during medical team rounds.

### Postintervention Phase

From February 24, 2014, to March 31, 2014, paired observations were performed daily on mechanically ventilated patients receiving sedative or analgesic medications as detailed in the baseline phase. After completion, research team members shared the scores with participating staff and used the time for education and discussion.

### Maintenance Phase

From May 4, 2014, to July 16, 2014, research team members and unit staff performed paired observation RASS assessments to evaluate the sustainability of the IRR.

### Statistical Analysis

Normally distributed demographic characteristics were presented using means and SDs. Medians and interquartile ranges were used for non-normally distributed clinical variables. Weighted-κ statistics and 95% confidence intervals evaluated agreement between research staff and PICU staff. Based on standard guidelines for substantial agreement, the goal weighted-κ was greater than 0.7.

## RESULTS

**Fig. 1.**
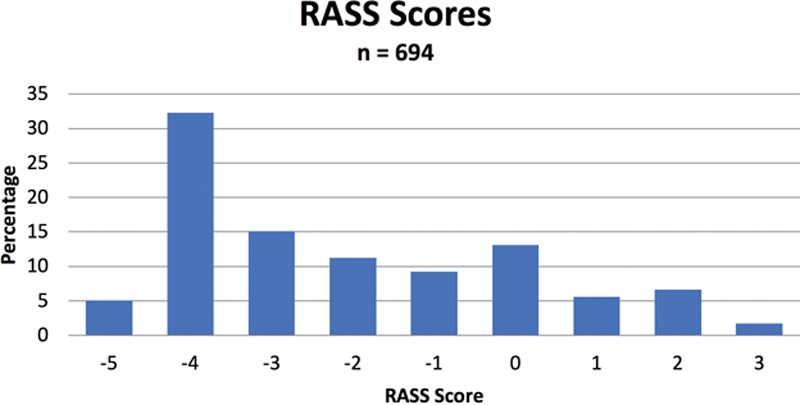
Distribution of RASS scores for 347 patient encounters.

Data were collected on a total of 45 patients, some of whom were evaluated multiple times during their ICU hospitalization. Table 2 displays the characteristics of the patient population included. Over all phases of the study, 73 nurses and 6 physicians completed a total of 347 paired assessments. The 694 RASS scores obtained ranged from −5 to +3, with a median score of −3. Seventy-three percentage of RASS scores were in the sedated range (−5 to −1), 13% were 0, and 14% were in the agitated range (+1 to +3). Figure 1 displays the distribution of RASS scores.

**Table 2.**
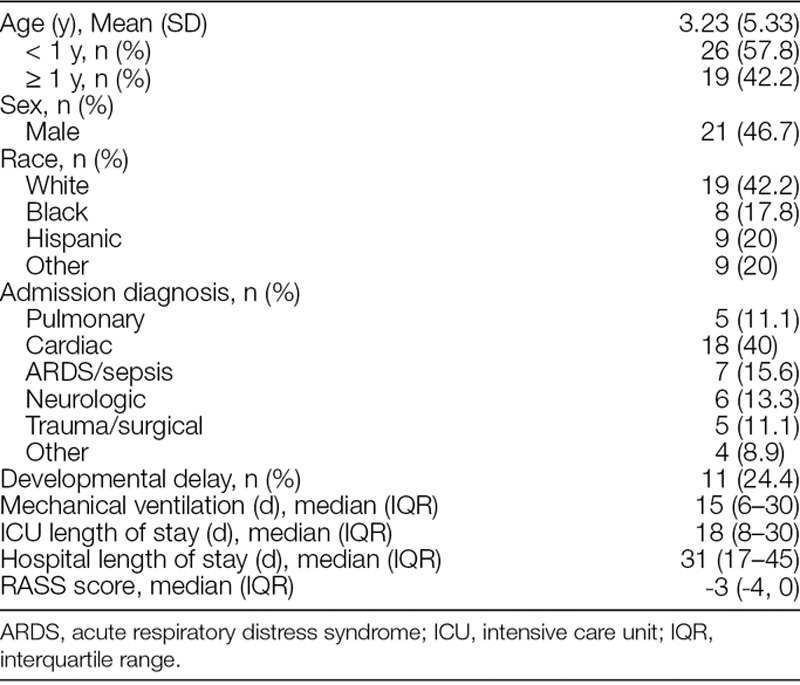
Demographic and Clinical Characteristics

### Baseline Phase

During the baseline phase, 16 nurses and 2 physicians completed a total of 49 paired observations on 20 patients. IRR was lower than expected, with a weighted κ of 0.56 (95% confidence interval, 0.39–0.72; Table 3). Reliability was lowest for nurse-physician paired assessments with a weighted κ of 0.18 and highest for physician–physician paired assessments at 0.88.

**Table 3.**
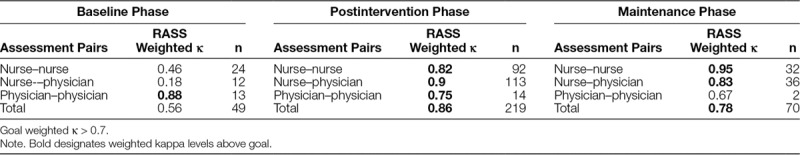
Inter-rater Reliability of the Richmond Agitation-sedation Scale

### Postintervention Phase

Over 90% of PICU staff completed the educational curriculum. During the postintervention phase, 36 nurses and 6 physicians completed a total of 228 paired observations on 15 patients. IRR improved significantly for all groups combined, with a weighted κ of 0.86 (95% confidence interval, 0.77–0.95; Table 3). The physician–physician weighted K decreased slightly (0.88–0.75).

### Maintenance Phase

In the maintenance phase, 21 nurses and 2 physicians completed a total of 70 paired observations on 11 patients. IRR maintained above goal during this phase, with a κ of 0.78 for all pairs combined (95% confidence interval, 0.61–0.94). The physician–physician weighted κ decreased slightly but was still near goal at 0.67. Analysis of subgroups revealed that in patients less than 1 year of age, the IRR improved from a weighted κ of 0.41 before intervention to 0.87 after the intervention. Similarly, in patients with developmental delay, the IRR improved from a weighted κ of 0.49 before intervention to 0.84 after the intervention.

## DISCUSSION

To our knowledge, this is the first investigation of an educational intervention to improve the IRR of the RASS in a pediatric population. The high weighted κ in the postintervention phase demonstrates the RASS to have the potential to be an extremely reliable tool for assessment of sedation status in mechanically ventilated, sedated PICU patients when a multi-modal educational curriculum is employed. Sustained IRR months after education and implementation ensure the feasibility of continued reliable use with time. Additionally, the IRR remained above goal despite an institutional transition to a new electronic medical record system between the postintervention and maintenance phases.

Previous studies demonstrate the utility of the RASS as an objective tool to assess sedation. However our experience did not reflect this as evidenced by poor reliability in the baseline phase despite the use of the RASS in daily sedation assessment by bedside nurses in the PICU for over 5 years. Several factors likely contributed. During the standard orientation, nurses received minimal training on how to correctly perform the RASS. Investigators noted the inconsistent use of the RASS by the medical team when describing sedation goals, varied nursing practice in the frequency of RASS assessment and documentation for mechanically ventilated patients, and difficulty in finding RASS documentation within the electronic medical record system. The implementation of a focused, multi-modal educational intervention by a multidisciplinary team was fundamental in improving the knowledge and utilization of the RASS with a subsequent significant improvement in IRR. Staff from multiple disciplines worked congruently to develop the various educational tools and to ensure each tool was appropriate for their colleagues. Multiple types of learning modules also accounted for different adult learning styles. One factor that was likely responsible for broad acceptance and utilization of the scale was the incorporation of after-evaluation feedback and debriefing sessions. These sessions identified opportunities for further education and discussion.

It is important that a sedation scale be reliable among differing intra- and inter-professional providers. Among nurse-to-nurse and nurse-to-physician pairs, there was a significant sustained improvement in RASS IRR after the educational intervention. However, the physician–physician pairs did not show similar improvement. Only the 2 physicians on the research team participated in the preintervention assessments, likely skewing the results and contributing to the high initial IRR compared with the postintervention results. Assessments in the postintervention and maintenance phases included both research and nonresearch physicians, thus contributing to slightly lower IRR scores. There were also only 2 physician–physician pairs in the maintenance phase assessments, potentially not demonstrating an accurate assessment of the IRR for this phase.

Importantly, the RASS can be a reliable tool in 2 unique patient populations frequently encountered in the PICU—children less than 1 year of age (weighted κ, 0.87) and developmentally delayed children (weighted κ, 0.84). The use of a slightly adapted version of the original RASS scale to include eye-opening, not just eye contact, for certain levels of sedation allowed the scale to be inclusive for patient populations in which the level of arousal is difficult to determine due to their developmental abilities.

There were several limitations to the study. First, the study evaluates use in a single institution. Also, several children required mechanical ventilation for a prolonged course during the postintervention phase. Thus, despite having over 200 RASS assessments, they were performed on a small number of patients. The timing of assessments on weekday shifts may have played a factor in the IRR. Many of the staff in the unit alternate between days and nights, but those working mainly nights represented a lower percentage of the RASS assessments obtained. Lastly, there was a paucity of assessments in the agitated range of the RASS. This result is largely due to the patient population included in the study as described previously. However, there were rare situations when research members were unable to complete RASS assessments on applicable patients due to too much activity in the room (eg, a patient undergoing a procedure or multiple visitors in the room).

## CONCLUDING SUMMARY

The RASS is a reliable scale for assessing sedation in pediatric patients after introduction of a multi-modal educational curriculum. Without appropriate training and education, the RASS should not be presumed to be a reliable scale among users. Ongoing education at least annually is likely important to ensure maintenance of knowledge, especially in units with high staff turnover. In the future, the educational framework used can be applied to improve the education of other tools utilized in critically ill pediatric patients.

## DISCLOSURE

The authors have no financial interest to declare in relation to the content of this article.
